# Outcomes of Antifungal Prophylaxis in High-Risk Haematological Patients (AML under Intensive Chemotherapy): The SAPHIR Prospective Multicentre Study

**DOI:** 10.3390/jof6040281

**Published:** 2020-11-12

**Authors:** Jean-Pierre Gangneux, Christophe Padoin, Mauricette Michallet, Emeline Saillio, Alexandra Kumichel, Régis Peffault de La Tour, Patrice Ceballos, Thomas Gastinne, Arnaud Pigneux

**Affiliations:** 1 Mycology Department, Centre Hospitalier Universitaire de Rennes, University Rennes, INSERM, Irset (Institut de Recherche en Santé, Environnement et Travail), UMR S_1085, 35000 Rennes, France; 2 Pharmacy Department, CHU Martinique Site P. Zobda Quitman, 97261 Fort de France, Martinique, France; christophe.PADOIN@chu-martinique.fr; 3 Clinical Haematology Department, Centre Léon Bérard (Anticancer Center), 28 Rue Laennec, 69373 Lyon, France; Mauricette.MICHALLET@lyon.unicancer.fr; 4 Department of Medical Affairs, MSD France, 10-12 cours Michelet, 92800 Puteaux, France; emeline.saillio@msd.com; 5 Scientific Department, ClinSearch, 110 Avenue Pierre Brossolette, 92240 Malakoff, France; alexandra.kumichel@clinsearch.net; 6 Haematology-Bone Marrow Transplant Department, Saint-Louis Hospital APHP, 1 Avenue Claude-Vellefaux, 75010 Paris, France; regis.peffaultdelatour@aphp.fr; 7 Clinical Haematology Department, CHRU Lapeyronie, 371 Avenue Doyen Gaston Giraud, 34295 Montpellier, France; p-ceballos@chu-montpellier.fr; 8 Clinical Haematology Department, CHU Nantes, 1 Place Alexis-Ricordeau, 44093 Nantes, France; thomas.gastinne@chu-nantes.fr; 9 Blood Diseases Department, Hospital Group Haut Leveque, Avenue de Magellan, 33604 Pessac, France; arnaud.pigneux@chu-bordeaux.fr

**Keywords:** haematological malignancies, invasive fungal disease, antifungal prophylaxis, neutropenia

## Abstract

Antifungal prophylaxis (AFP) is recommended by international guidelines for patients with acute myeloid leukaemia (AML) undergoing induction chemotherapy and allogeneic hematopoietic cell transplantation. Nonetheless, treatment of breakthrough fungal infections remains challenging. This observational, prospective, multicentre, non-comparative study of patients undergoing myelosuppressive and intensive chemotherapy for AML who are at high-risk of invasive fungal diseases (IFDs), describes AFP management and outcomes for 404 patients (65.6% newly diagnosed and 73.3% chemotherapy naïve). Ongoing chemotherapy started 1.0 ± 4.5 days before inclusion and represented induction therapy for 79% of participants. In 92.3% of patients, posaconazole was initially prescribed, and 8.2% of all patients underwent at least one treatment change after 17 ± 24 days, mainly due to medical conditions influencing AFP absorption (65%). The mean AFP period was 24 ± 32 days, 66.8% stopped their prophylaxis after the high-risk period and 31.2% switched to a non-prophylactic treatment (2/3 empirical, 1/3 pre-emptive/curative). Overall, 9/404 patients (2.2%) were diagnosed with probable or proven IFDs. During the follow-up, 94.3% showed no signs of infection. Altogether, 20 patients (5%) died, and three deaths (0.7%) were IFD-related. In conclusion, AFP was frequently prescribed and well tolerated by these AML patients, breakthrough infections incidence and IFD mortality were low and very few treatment changes were required.

## 1. Introduction

Invasive fungal diseases (IFDs) are the most important cause of morbidity and mortality in patients with haematological malignancies, especially among those with neutropenia induced by chemotherapy and recipients of allogeneic hematopoietic stem cell transplants (HSCT) [1,2]. In recent years, with the increased use of intensive chemotherapy and immunosuppressive agents, as well as HSCT, IFD prevalence has increased dramatically [3,4]. In patients with haematological conditions, the number of probable or proven IFDs within the last two decades varied between 3% and 13%, with a reported mortality varying from 10% to 57% [5,6,7,8,9]. In this context, invasive candidiasis and aspergillosis account for more than 90% of these infections [10]. Publications of clinical studies and autopsy findings indicate that IFDs have the highest incidence in patients suffering from acute myeloid leukaemia (AML) and myelodysplastic syndromes (MDS) [3,11,12]. 

IFD classification, according to the international consensus criteria of the “Invasive Fungal Infection Group” of the “European Organisation for Research and Treatment of Cancer” (EORTC) and the “Mycoses Study Group” (MSG), is based on the level of diagnostic certainty and includes proven, probable or possible infection [13,14]. An early diagnosis and immediate initiation of a systematic antifungal (AF) therapy has been demonstrated to substantially improve outcomes [9,15,16]. Nevertheless, observations of clinical practice show that the diagnosis and management of infected patients with IFDs remains challenging. Thus, IFD prevention is now a major objective in the management of patients with haematological malignancies. The introduction of antifungal prophylaxis (AFP) for high-risk patients in combination with environmental protection has markedly reduced the overall incidence of IFD, demonstrating that AFP is an important strategy in the clinical care of haematological patients [2,11,17,18,19,20]. 

Despite the publication of frequently updated recommendations and guidelines [21,22,23], a certain number of patients develop breakthrough IFDs, which are associated with substantial mortality [24,25,26]. The described morbidity and mortality of breakthrough IFDs are frequently attributed to emerging and often drug-resistant atypical pathogens, indicating microbial shifts in colonization [12,23,27,28] or the non-exhaustive coverage of patient monitoring during AFP, especially regarding diagnostic methods of monitoring fungal infection. 

In France, IFDs account for a major part of the fungal burden in immunosuppressed patients [29]. Little has been published regarding the management of patients with haematological conditions at high-risk of IFDs since the significant changes related to published guidelines and AFP prescription [9,20,30].

Therefore, the present study aimed to provide an extensive, diagnostic and therapeutic description of patients at high risk of IFD under AFP in French haematological services in order to evaluate outcomes and potential benefits. 

## 2. Patients and Methods

### 2.1. Ethics

The study was performed in accordance with the Declaration of Helsinki as well as national and institutional standards. According to French regulatory requirements, approvals were obtained from the French review boards (Comité Consultatif sur le Traitement de l’Information en Matière de Recherche dans le Domaine de la Santé, Commission Nationale Informatique et Liberté—DR-2015-629 obtained 30 December 2015). All patients were informed about the study and provided their oral consent prior to inclusion. 

### 2.2. Study Design and Patients

**Figure 1:**
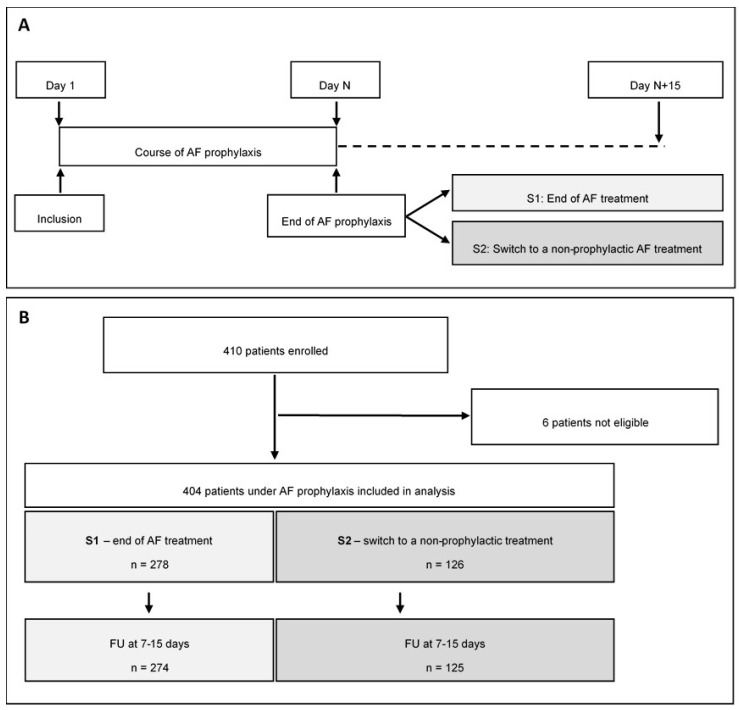
(**A**) Study flow-chart; (**B**) Patient flow-chart.

This was an observational, multicentre, prospective study conducted in 23 French haematology centres between June 2016 and November 2018. The participating centres were selected based on their experience with AML treatment, (treatment of >10 AML patients per year). Enrolment was limited to adult patients (≥18 years), presenting, or susceptible to present, a profound and prolonged neutropenia in the context of a myelosuppressive intensive chemotherapy for AML and initiating a systemic primary AFP. Participants were recruited upon AFP prescription. The follow-up period started from AFP initiation and ended 15 days after the end of prophylaxis (Figure 1A). 

### 2.3. Data Source and Statistical Analysis

Investigators entered the patients’ data in electronic case report forms (eCRFs) including the history of the underlying disease, the medical as well as hospitalisation conditions and the AF prophylactic and, if relevant, non-prophylactic treatments. Furthermore, clinical signs, X-ray exams and mycological tests, such as blood cultures, galactomannan antigen tests, PCRs and mycological cultures related to IFD episodes were reported for any patient having undergone pre-emptive, empirical or curative treatment. A quality control for the recorded data was performed in 10% of the active participating centres (five patients per centre). 

The statistical analyses for this French real-life study were mainly descriptive. The quantitative variables were described by the number of patients with available data. Of those, mean, standard deviation (SD), median and range were calculated. Qualitative variables were also described by the number of available data. Frequencies were reported as percentages for each category and 95% confidence intervals (CIs) were computed for relevant parameters. Additional subgroup analyses were performed for subgroups S1 (definitive end of the AF prophylactic treatment) and S2 (prophylaxis discontinuation and switch to a non-prophylactic AF treatment). The definitive end of AFP was decided for patients who were at the end of their high-risk period (Absolute Neutrophil Count [ANC] > 0.5 G/L) and had no signs or symptoms of infection. Introduction of a non-prophylactic AF treatment was either empirical, pre-emptive or curative and the decision was based on persistent fever, signs and symptoms of infection or positive results from blood cultures, mycological exams, antifungal sensitivity tests and/or imaging. Pearson’s chi-squared test and Student’s *t*-test were used to compare discrete and continuous variables, respectively. Multivariate logistic regression analyses with backward selection was performed on the parameters showing a significant difference between the two subgroups during baseline, treatment initiation, end of treatment and 15-day follow up.

## 3. Results

### 3.1. Baseline Demographics, Medical History and Medical Condition

The 23 participating French haematology departments enrolled 410 patients, of which 404 were eligible and included in the data analysis (Figure 1B). Patient baseline characteristics are presented in Table 1. The mean age was 56.4 ± 14.0 years and 207 (51.2%) participants were men. The mean duration between AML diagnosis and study inclusion/AFP treatment initiation was 64.9 ± 178 days. Two thirds (*n* = 265; 65.6%) of the population suffered from a newly diagnosed haematological malignancy and, according to cytogenetics and molecular prognosis factors, most patients (*n* = 162; 40.6%) were classified as “intermediate”. Among the entire population, 296 (73.3%) patients were chemotherapy naïve. The ongoing chemotherapy began 1.0 ± 4.5 days before inclusion and was an induction chemotherapy for 328 participants (81.2%) consolidation for 44 patients (10.9%) and relapse chemotherapy for 32 patients (7.9%). 

During data analysis, the 404 patients were classified in two subgroups. S1 was comprised of 278 (68.8%) patients who took AFP until the end of their high-risk period, while the 126 (31.2%) S2 patients discontinued AFP and switched to a non-prophylactic AF treatment. 

The patients’ medical and treatment history are summarised in Table 1. Comparison of the subgroups showed significant differences concerning the patient’s genetic factors such as cytogenetics and molecular markers; AML onset; any previous chemotherapy treatments; and the nature and the duration of the ongoing chemotherapy before inclusion. The two major IFD risk factors at baseline were neutropenia (Absolute Neutrophil Count (ANC) < 0.5 G/L), which started 11.0 ± 29.7 days before inclusion and affected 312 patients (77.2%) and the patients’ advanced age (patients ≥ 65 years) (*n* = 134; 33.2%) (Table 2).

Slightly more than half (53.3%) of the population experienced a medical condition potentially impacting AFP absorption. A total of 196 patients (48.5%) took gastric cytoprotectants and/or proton pump inhibitors (PPIs) and 387 patients (95.8%) continued to eat. The majority of the latter received protected (51.8%) or sterile (42.5%) food. Most patients (93.5%) were treated in a sterile/isolated area and 144 (35.8%) received digestive decontamination, which was either antibacterial (*n* = 93), antifungal (*n* = 3) or both (*n* = 48). The patients’ IFD risk factors and baseline medical conditions are summarised in Table 2.

### 3.2. Initial AFP Treatment and Modifications during the Progression of the Disease

**Figure 2:**
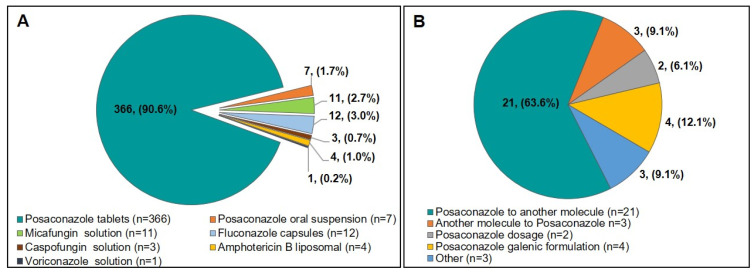
(**A**) Initially prescribed molecules for antifungal prophylaxis (**B**) First antifungal prophylaxis modification per patient.

AFP treatments were initially prescribed according to the hospital’s own specific protocol for 389 patients (96.8%), and most of the patients (*n* = 373, 92.3%) received posaconazole, either as tablets (*n* = 366, 90.6%) or as oral suspension (*n* = 7, 1.7%), (Figure 2A). A loading dose was given to 283 participants (70.2%), with a significant difference between the two subgroups (S1: *n* = 205, 73.5% vs. S2: *n* = 78, 62.9%; *p* = 0.032) (Table 3). Therapeutic drug monitoring (TDM) was performed at half (12/23) of the participating centres and posaconazole plasma concentrations (PPCs) were available for 139 patients with 1 to 10 measurements taken per patient. The mean PPC, derived from measurements of 139 patients in 12 centres, was 1.2 ± 0.9 mg/L with a significant difference between the two subgroups. However, this was mainly due to the data of a single patient in S2 for whom 10 measures with high concentrations were taken. The first measurement per patient, which is a more relevant item, was performed 9.1 ± 7.2 days after initiation with a mean PPC of 1.1 ± 0.7 mg/L. Among these, 40/139 (28.8%) patients displayed PPCs below the target concentration of 0.7 mg/L with no statistically significant differences between the two subgroups (S1: 27.7% vs. S2: 30.4%, *p* = 0.735) (Table 4). 

During their prophylactic treatment phase, only 33 patients (8.2%) underwent prescription changes, with the first change occurring 17.3 ± 23.9 days after inclusion. Forty prescription changes were made in total for the 33 patients. For 21 of these patients this was a switch from posaconazole to another molecule, while among the 13 remaining patients, six changed posaconazole dosage or galenic formulation, four switched from another molecule to posaconazole and the data of the two last patients were incomplete (Figure 2B). PPCs were measured for 15/33 patients and reached 0.9 ± 0.6 mg/L while the mean concentration of the 124 patients without treatment modifications was 1.2 ± 0.9 mg/L (Table 4). The initial AFP treatment and the modifications undertaken during the study are summarised in Table 3, while the results of the TDM are presented in Table 4.

### 3.3. End of AFP Treatment 

**Figure 3:**
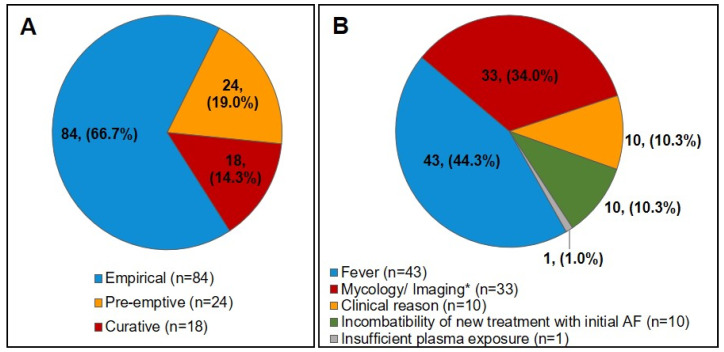
(**A**) Treatment introduced after the end of the antifungal prophylactic treatment; (**B**) Reasons for introduction of a non-prophylactic treatment; * Mycology and imaging tests include: blood culture, mycological examinations, antigen detection, antifungal sensitivity test

The mean AFP period was 24.2 ± 32.1 days after which the majority of the population (*n* = 267, 66.8%) stopped the prophylactic treatment due to the end of their high-risk period. AFP duration in S1 was longer than in S2, with 26.8 ± 33.4 days versus 18.6 ± 28.1 days, respectively. The PPCs at the end of the AFP (last dose of AFP ±2 days) were available for 20 patients in S1 and 30 patients in S2 and reached 1.5 ± 0.9 mg/L and 1.3 ± 1.0 mg/L, respectively (*p* = 0.586) (Table 4). Of the 126 patients who switched to a non-prophylactic AF treatment, 84 (66.7%) received an empirical, 24 (19.0%) a pre-emptive and 18 (14.3%) a curative AF therapy (Figure 3A). The decision to introduce a curative AF treatment for 18/404 patients (4.5%) was based on positive results from blood cultures, mycological exams, antifungal sensitivity tests and/or imaging (Figure 3B). Among those, 9/404 patients (2.2%) were diagnosed with probable or proven IFDs including five probable invasive aspergillosis, two pulmonary pneumocystosis, one mucor-mycosis and one fungemia caused by *Saccharomyces* sp. Other treatment changes were based on clinical signs (mainly fever), biological tests and/or imaging, as summarized in Table 5. 

At the end of the AFP, 256 participants had presented a medical condition which could potentially have influenced AFP absorption: 164 patients (59.2%) in S1 and 92 (73.0%) in S2, (*p* = 0.008). PPCs were available for 14 patients in S1 (1.5 ± 1.0 mg/L) and 23 patients in S2 (1.2 ± 1.0 mg/L) (Table 4). In total, 211 patients (52.4%) took gastric cytoprotectants and/or PPIs and 347 (86.5%) were able to eat. Concerning the last patients, 102 (29.5%) received sterile, 136 (39.3%) protected and 108 (31.2%) normal food, respectively. Surprisingly, more S1 than S2 patients received normal food (S1: *n* = 104, 41.8% vs. S2: *n* = 4, 4.1%; *p* < 0.001). Digestive decontamination was used for 92 patients (22.9%) and for 24 patients (6.0%) hospitalisation conditions had changed, while 96 patients were no longer hospitalised. Patients’ medical conditions at the end of AF prophylaxis are summarised in Table 5 and the results of the TDM are presented in Table 4.

### 3.4. Patient Status 15 Days after the End of AFP Treatment and Deaths

At the 7–15 day follow-up visit, data were available for 387 patients. Among them, 22 (5.7%) exhibited signs and/or symptoms of infection, with 2 and 20 patients in S1 and S2, respectively (*p* < 0.001). 

Of the 404 patients in the total study population, 91.6% (*n* = 370) experienced periods of profound neutropenia (ANC < 0.5 G/L), with a mean of 1.1 ± 0.5 occurrences per patient and a significant statistical difference upon subgroup comparison (S1: 1.0 ± 0.3 vs. S2: 1.2 ± 0.8; *p* = 0.021). Furthermore, 20 patients died during the course of the study, after a mean duration of 57.6 ± 50.3 days following inclusion. Of those, the death of three patients who were in group S2, was directly related to an IFDs: two to invasive aspergillosis, and one to fungemia caused by *Saccharomyces* sp. Only one had benefited from a curative therapy and two received a pre-emptive treatment. Furthermore, haematological status was not favourable for all three: two patients with secondary haematological malignancies and one newly diagnosed AML with unfavourable cytogenetics prognosis. The patients’ status at day 15, profound neutropenia periods and deaths are summarised in Table 6.

### 3.5. Analyses of Significantly Different Parameters between the Subgroups

A multivariate logistic regression identified several parameters associated with the switch to a non-prophylactic AF treatment. The analyses revealed that patients with an unfavourable cytogenetics/molecular biology prognosis, chemotherapy naïve patients and those who had not received an AFP loading dose were more likely to switch in subgroup 2. An increasing duration between the start of the ongoing chemotherapy and the AFP initiation were also associated with the introduction of the non-prophylactic AF treatment. 

Regarding the analysed parameters after the end of the AFP treatment, the results clearly indicate an association between the patients’ alimentation (patient cannot eat, but oral medication intake and reception of protected food are possible) with the treatment switch. Further associations were identified between the existence of signs/symptoms of infection as well as the patients’ hospitalization conditions at follow-up and the number of profound neutropenia periods. The results of these analyses are summarised in Table 7.

## 4. Discussion

The SAPHIR study was the first French prospective observational study to describe the outcomes of AFP in terms of management and follow up of haematology patients at high-risk of IFD. The incidence of breakthrough IFDs and deaths attributable to IFD have been described among this particularly fragile population.

Broad-spectrum triazoles are considered the current standard of care for AFP during the treatment of patients receiving myelosuppressive intensive chemotherapy for AML or MDS. The majority of the study population received posaconazole (92%) as their primary AFP, consistent with ECIL-5 and ESCMID-ECMM-ERS recommendations, as well as previously published studies [23,31]. In their landmark study, Cornely et al. demonstrated more efficient prevention of IFDs with the use of posaconazole than with fluconazole or itraconazole, and an increased overall survival of posaconazole treated patients undergoing induction chemotherapy for AML or MDS [2]. In another study, Vehreschild et al. showed a decreased IFD incidence rate, number of febrile days and hospitalisation duration upon the introduction of posaconazole prophylaxis in AML patients undergoing first remission-induction chemotherapy [32]. 

The internationally recommended prophylactic posaconazole plasma concentration of >0.7 mg/L is more easily achieved when using the tablet formulation compared to the oral suspension. Moreover, the tablet formulation is associated with low levels of inter-individual variability and therefore routine therapeutic drug monitoring is not recommended [22,33]. As such, only half of the participating centres performed posaconazole TDM in a third of the enrolled patients. The first measurements were taken after an average of nine days from treatment initiation and in our study no significant differences were seen between the two subgroups regarding the proportion of patients below the target concentration of 0.7 mg/L. 

In addition, our results indicate that the patient’s conditions potentially influencing AFP absorption at the end of the prophylactic treatment phase do not affect the need for a non-prophylactic AF treatment, which is consistent with previously published data and confirm the good biodisponibility of posaconazole [34,35]. However, the number of PPC measurements available throughout the study is small and allows only limited conclusions to be drawn. 

The relatively low number of patients (33/404) having undergone AFP treatment modifications during this trial, including the few measured PPCs below the target of 0.7 mg/L, indicate a high tolerability and acceptability of the molecules prescribed, and is similar to previously published studies [2,36]. Of note, the multivariate analysis underlines that patients with an unfavourable cytogenetics/molecular biology prognosis and chemotherapy naïve patients were more likely to switch to empirical/curative treatment.

Another interesting finding concerns the 283 patients who received a loading dose of the prescribed AFP and were observed to be less likely to receive a non-prophylactic treatment, implying a more efficient protection from IFDs. Thus, the administration of a loading dose at treatment initiation appears to have enabled posaconazole therapeutic concentrations to be reached more rapidly (*p* = 0.032; OR:1.811 [1.116–2.938]. However, due to the absence of systematic TDM by all participating centres, this could not be explored further. 

One of the major reported complications in the AFP treatment of AML patients is the development of breakthrough IFDs. The incidence of probable/proven breakthrough IFDs in patients under AFP reported herein was 2.2%, and the overall IFD-related mortality was 0.7%. These results are consistent with previously published real-life data on AML patients undergoing intensive chemotherapy. In a retrospective study, Rodríguez-Veiga et al. reported 4.8% of probable/proven IFDs in 285 subjects with 589 episodes of intensive chemotherapy under AFP with fluconazole, itraconazole and voriconazole [37]. Vehreschild et al. retrospectively analysed 159 AML patients under AFP and demonstrated a probable/proven IFD incidence of 3.9% with posaconazole versus 19.5% with topic polyenes [32]. 

Clinical trials involving more heterogeneous cohorts with different haematological malignancies also found similar results. The randomised study conducted by Cornely et al. analysed 602 patients with AML or MDS who received either posaconazole or fluconazole/itraconazole. The reported incidence of probable/proven IFDs was 2% in the posaconazole group and 8% in the fluconazole or itraconazole group. The retrospective study of 174 patients with AML or MDS performed by Tormo et al. reported 1.7% probable/proven IFDs in the posaconazole group and 5.3% in the itraconazole group [38]. However, direct comparison of breakthrough IFDs between different clinical trials should be carefully considered due to the lack of consensus definitions for breakthrough IFDs. Cornely et al. recently proposed corresponding definitions to support future study design and to harmonise and facilitate data interpretation accordingly [28]. 

We observed that patients in the S2 group (those that received a non-prophylactic AF treatment) underwent induction chemotherapy significantly more often than those in the S1 group (receiving only a prophylactic treatment) (88.9% vs. 74.5%). These results are similar to data reported by Pagano et al. who observed invasive aspergillosis predominantly in those AML patients with post-induction aplasia [3,11]. This is probably due to the patients’ pre-hospital exposure to fungi and tissue damage, and particularly mucositis and gastrointestinal malabsorption caused by the intensive chemotherapy regimen.

The majority of patients who switched to a non-prophylactic therapy after AFP discontinuation, received an empirical treatment (66.7%), with the medical decision having been taken mainly due to persistent fever. However, during the 15 days follow-up, none of the fever-related empirical episodes were shown to be attributed to IFDs. This observation further intensifies the controversy over premature switches from prophylactic to empiric AF treatments. Current guidelines recommend an empirical AF treatment in high-risk neutropenia patients with resistant or recurrent fevers [21,31]; however, with improved IFD diagnostic testing, pre-emptive AF therapy could become a better alternative to the empirical approach, reducing the risk of emerging resistance, as well as limiting costs and exposure to any AF treatment toxicities. Results from several studies suggest that pre-emptive therapy in patients with haematological malignancies leads to decreased AF treatment duration and rates without raising IFD-related mortality [6,39]. All these results indicate that a considerable number of high-risk patients under AFP develop fever from a non-fungal origin, which has yet to be thoroughly investigated.

To minimize the risk of IFDs, high-risk haematological patients are conventionally treated in a protected environment including isolation units with HEPA filtration systems, sterile food and the use of antibacterial and/or antifungal gut decontamination. The benefits of such protected environments in reducing infection incidence was discussed by Bodey et al. and Gangneux et al. and has been introduced into European ESCMID-ECMM-ERS guidelines and national guidelines [23,40,41]. Although its efficacy is debated, in our study 93.5% of patients were treated in sterile/isolated areas including portable air flow, laminar air flow and/or HEPA filter, demonstrating the routine use of a combination of AFP treatment and environmental measures, which are undertaken given the very high IFD risk for AML patients undergoing intensive chemotherapy.

Potential prognostic factors for the necessity of a switch from AFP to a non-prophylactic treatment were also identified. Subgroup analysis indicated that those patients with more favourable cytogenetic profiles or molecular biology prognoses required less non-prophylactic AF therapy. Furthermore, patients undergoing chemotherapy for relapse, consolidation or who had undergone a previous chemotherapy received fewer non-prophylactic treatments after discontinuation of AFP.

This study does have certain limitations. (1) As participation in this study was voluntary, the selected centres might not be entirely representative of AFP practice throughout France which may have led to a certain selection bias. (2) Moreover, the observational study design was based on real-life clinical practice of the centres and hence explains the absence of systematic drug monitoring. These results provide an extensive and informative overview of real-life diagnostic and therapeutic IFD management practices in a large sample of AML patients receiving intensive chemotherapy and AFP at 23 specialized French centres.

## Figures and Tables

**Table 1:** Baseline characteristics.

Characteristics	Switch to a Non-Prophylactic Treatment			
	No (S1)	Yes (S2)	Total	*p*-Value
Age (years)	*n* = 278	*n* = 126	*n* = 404	0.273
Mean (SD)	55.9 (14.8)	57.6 (12.2)	56.4 (14.0)	
Median	59.5	60.5	60.0	
Range	19.0–88.0	21.0–78.0	19.0–88.0	
Gender	*n* = 278 (%)	*n* = 126 (%)	*n* = 404 (%)	0.340
Male	138 (49.6)	69 (54.8)	207 (51.2)	
Medical and treatment history				
Time since AML diagnosis (days)	*n* = 263	*n* = 116	*n* = 379	0.823
Mean (SD)	66.0 (152)	62.3 (227)	64.9 (178)	
Median	10.0	7.5	9.0	
Range	1.0–1256	1.0–1737	1.0–1737	
Cytogenetics/molecular biology of prognosis	*n* = 274 (%)	*n* = 125 (%)	*n* = 399 (%)	0.001
Favourable	87 (31.8)	22 (17.6)	109 (27.3)	
Intermediate	108 (39.4)	54 (43.2)	162 (40.6)	
Unfavourable	59 (21.5)	45 (36.0)	104 (26.1)	
Do not know	20 (7.3)	4 (3.2)	24 (6.0)	
AML onset	*n* = 278 (%)	*n* = 126 (%)	*n* = 404 (%)	0.004
Newly diagnosed	177 (63.7)	88 (69.8)	265 (65.6)	
Relapsed	20 (7.2)	8 (6.4)	28 (6.9)	
Refractory	2 (0.7)	2 (1.6)	4 (1.0)	
In consolidation	40 (14.4)	4 (3.2)	44 (10.9)	
Secondary	39 (14.0)	24 (19.0)	63 (15.6)	
Previous chemotherapy	*n* = 278 (%)	*n* = 126 (%)	*n* = 404 (%)	0.001
No	190 (68.3)	106 (84.1)	296 (73.3)	
Chemotherapy at baseline				
Time since start of ongoing chemotherapy (days)	*n* = 277	*n* = 125	*n* = 402	0.001
Mean (SD)	−1.6 (4.7)	0.03 (3.9)	−1.0 (4.5)	
Median	1.0	1.0	1.0	
Range	−16.0–16.0	−13.0–10.0	−55.0–29.0	
Ongoing chemotherapy	*n* = 278 (%)	*n* = 126 (%)	*n* = 404 (%)	0.003
Consolidation	40 (14.4)	4 (3.2)	44 (10.9)	
Induction	216 (77.7)	112 (88.9)	328 (81.2)	
Relapse	22 (7.9)	10 (7.9)	32 (7.9)	

AML: acute myeloid leukaemia.

**Table 2:** IFD risk factors and baseline medical conditions.

Characteristics	Switch to a Non-Prophylactic Treatment			
	No (S1)	Yes (S2)	Total	*p*-Value
Risk factors				
Neutropenia	*n* = 278 (%)	*n* = 126 (%)	*n* = 404 (%)	0.344
Yes	211 (75.9)	101 (80.2)	312 (77.2)	
Advanced age	*n* = 278 (%)	*n* = 126 (%)	*n* = 404 (%)	0.857
Yes	93 (33.5)	41 (32.5)	134 (33.2)	
Monocytopenia	*n* = 278 (%)	*n* = 126 (%)	*n* = 404 (%)	0.116
Yes	46 (16.5)	18 (14.3)	64 (15.8)	
Absence of air filtration by high efficiency particulate air (HEP)filter	*n* = 278 (%)	*n* = 126 (%)	*n* = 404 (%)	0.046
Yes	17 (6.1)	15 (11.9)	32 (7.9)	
Baseline medical conditions				
Time to neutropenia start (days)	*n* = 206	*n* = 100	*n* = 306	0.314
Mean (SD)	9.8 (25.7)	13.4 (36.5)	11.0 (29.7)	
Median	4.0	4.5	4.0	
Range	−10.0–289	−5.0–305	−10.0–305	
Medical condition potentially influencing AFP absorption	*n* = 277 (%)	*n* = 126 (%)	*n* = 403 (%)	0.303
Yes	143 (51.6)	72 (57.1)	215 (53.3)	
Gastric cytoprotectants and/ or PPI	*n* = 278 (%)	*n* = 126 (%)	*n* = 404 (%)	0.537
Yes	132 (47.5)	64 (50.8)	196 (48.5)	
Patient can eat	*n* = 278 (%)	*n* = 126 (%)	*n* = 404 (%)	1.000
Yes	266 (95.7)	121 (96.0)	387 (95.8)	
No, oral medication intake possible	11 (4.0)	5 (4.0)	16 (4.0)	
No, oral medication intake impossible	1 (0.4)	-	1 (0.3)	
If able to eat, patient receives food:	*n* = 265 (%)	*n* = 121 (%)	*n* = 386 (%)	0.274
Sterile	115 (43.4)	49 (40.5)	164 (42.5)	
Protected	132 (49.8)	68 (56.2)	200 (51.8)	
Normal	18 (6.8)	4 (3.3)	22 (5.7)	
Method of digestive decontamination	*n* = 276 (%)	*n* = 126 (%)	*n* = 402 (%)	0.068
Yes	107 (38.8)	37 (29.4)	144 (35.8)	
Type of digestive decontamination	*n* = 107 (%)	*n* = 37 (%)	*n* = 144 (%)	0.513
Antibacterial	67 (62.6)	26 (70.3)	93 (64.6)	
Antifungal	2 (1.9)	1 (2.7)	3 (2.1)	
Antibacterial + Antifungal	38 (35.5)	10 (27.0)	48 (33.3)	
Hospitalisation conditions	*n* = 276 (%)	*n* = 126 (%)	*n* = 402 (%)	0.070
Conventional area	22 (8.0)	4 (3.2)	26 (6.5)	
Sterile/isolated area	254 (92.0)	122 (96.8)	376 (93.5)	
If sterile/isolated area	*n* = 254 (%)	*n* = 122 (%)	*n* = 376 (%)	0.110
Portable air treatment	53 (20.9)	17 (13.9)	70 (18.6)	
Laminar air flow	50 (19.7)	33 (27.0)	83 (22.1)	
HEPA filter	95 (37.4)	52 (42.6)	147 (39.1)	
Laminar air flow and HEPA filter	56 (22.0)	20 (16.4)	76 (20.2)	

IFD: invasive fungal disease; AFP: antifungal prophylaxis; PPI: proton pump inhibitor.

**Table 3:** Initial AFP treatment and modifications.

Characteristics	Switch to a Non-Prophylactic Treatment			
	No (S1)	Yes (S2)	Total	*p*-Value
Reason(s) for AFP prescription	*n* = 276 (%)	*n* = 126 (%)	*n* = 402 (%)	0.784
Hospital specific protocol	268 (97.1)	121 (96.0)	389 (96.8)	
Link to medical history	3 (1.1)	1 (0.8)	4 (1.0)	
Both	5 (1.8)	4 (3.2)	9 (2.2)	
Loading dose	*n* = 279 (%)	*n* = 124 (%)	*n* = 403 (%)	0.032
Yes	205 (73.5)	78 (62.9)	283 (70.2)	
AFP modification	*n* = 278 (%)	*n* = 126 (%)	*n* = 404 (%)	0.369
Yes	25 (9.0)	8 (6.3)	33 (8.2)	
Time to first modification (days)	*n* = 25	*n* = 6	*n* = 31	0.915
Mean (SD)	17.5 (25.5)	16.3 (17.8)	17.3 (23.9)	
Median	12.0	10.0	11.0	
Range	0.0–108	2.0–51	0.0–108	

AFP: antifungal prophylaxis.

**Table 4:** Posaconazole therapeutic drug monitoring during the study.

Characteristics	Switch to a Non-Prophylactic Treatment			
	No (S1)	Yes (S2)	Total	*p*-Value
Global PPC measurements (mg/L)				
Global PPC of all measurements taken	*n* = 172	*n* = 105	*n* = 277	0.027
Mean (SD)	1.1 (0.7)	1.3 (1.1)	1.2 (0.9)	
Median	0.9	1.0	1.0	
IQR	0.6–1.3	0.6–1.8	0.6–1.6	
Duration to 1st PPC per patient (days)	*n* = 83	*n* = 56	*n* = 139	0.148
Mean (SD)	9.9 (6.5)	8.1 (8.1)	9.1 (7.2)	
Median	7.0	6.0	7.0	
Range	3.0–33	0.0–48	0.0–48	
First PPC per patient	*n* = 83	*n* = 56	*n* = 139	0.420
Mean (SD)	1.0 (0.6)	1.1 (0.8)	1.1 (0.7)	
Median	0.9	1.0	1.0	
IQR	0.6–1.3	0.5–1.6	0.6–1.3	
Patients with PPC < 0.7 mg/L	*n* = 83 (%)	*n* = 56 (%)	*n* = 139 (%)	0.735
	23 (27.7)	17 (30.4)	40 (28.8)	
AFP modification during the study				
Patient changes AFP	No (*n* = 124)	Yes (*n* = 15)	Total (*n* = 139)	*p*-value
PPC (mg/L) of all measurements taken	*n* = 252	*n* = 25	*n* = 277	0.189
Mean (SD)	1.2 (0.9)	0.9 (0.6)	1.2 (0.9)	
Median	1.0	0.8	1.0	
IQR	0.6–1.6	0.5–1.2	0.6–1.6	
Characteristics	Switch to a Non-Prophylactic Treatment			
	No (S1)	Yes (S2)	Total	*p*-value
PPC measurements at end of AFP (mg/L)				
PPC at end of AFP	*n* = 20	*n* = 30	*n* = 50	0.586
Mean (SD)	1.5 (0.9)	1.3 (1.0)	1.4 (1.0)	
Median	1.2	1.1	1.1	
IQR	0.7–2.0	0.5–2.0	0.7–2.0	
Patients with PPC < 0.7 mg/L	*n* = 20 (%)	*n* = 30 (%)	*n* = 50 (%)	0.430
Yes	4 (20.0)	9 (30.0)	13 (26.0)	
PPC in patients with potential AFP absorption issues	*n* = 14	*n* = 23	*n* = 37	0.373
Mean (SD)	1.5 (1.0)	1.2 (1.0)	1.3 (1.0)	
Median	1.2	0.8	1.0	
IQR	0.7–2.0	0.4–1.7	0.6–1.9	
Patients with PPC < 0.7 mg/L	*n* = 14 (%)	*n* = 23 (%)	*n* = 37 (%)	0.477
Yes	3 (21.4)	8 (34.8)	11 (29.7)	

AFP: anti-fungal prophylaxis; IQR: interquartile range; PPC: posaconazole plasma concentration.

**Table 5:** End of antifungal prophylaxis.

Characteristics	Switch to a Non-Prophylactic Treatment			
	No (S1)	Yes (S2)	Total	*p*-Value
Duration of prophylaxis period (days)	*n* = 277	*n* = 126	*n* = 403	0.017
Mean (SD)	26.8 (33.4)	18.6 (28.1)	24.2 (32.1)	
Median	22.0	13.0	19.0	
Range	1.0–375	1.0–209	1.0–375	
Medical conditions influencing AFP absorption				
Medical condition potentially influencing AFP absorption	*n* = 277 (%)	*n* = 126 (%)	*n* = 403 (%)	0.008
Yes	164 (59.2)	92 (73.0)	256 (63.5)	
Gastric cytoprotectants and/or PPI	*n* = 277 (%)	*n* = 126 (%)	*n* = 403 (%)	0.386
Yes	141 (50.9)	70 (55.6)	211 (52.4)	
Patient can eat	*n* = 275 (%)	*n* = 125 (%)	*n* = 401 (%)	0.001
Yes	250 (90.9)	97 (77.0)	347 (86.5)	
No, oral medication intake possible	16 (5.8)	19 (15.1)	35 (8.7)	
No, oral medication intake impossible	9 (3.3)	10 (7.9)	19 (4.7)	
If able to eat, patient receives food:	*n* = 249 (%)	*n* = 97 (%)	*n* = 346 (%)	<0.001
Sterile	69 (27.7)	33 (34.0)	102 (29.5)	
Protected	76 (30.5)	60 (61.9)	136 (39.3)	
Normal	104 (41.8)	4 (4.1)	108 (31.2)	
Method of digestive decontamination used	*n* = 275 (%)	*n* = 126 (%)	*n* = 401 (%)	0.070
Yes	56 (20.4)	36 (28.6)	92 (22.9)	
Type of digestive decontamination				
Antibacterial	50 (89.3)	33 (91.7)	83 (90.2)	
Antifungal	13 (23.2)	4 (11.1)	17 (18.5)	
Change of hospitalisation conditions	*n* = 275 (%)	*n* = 126 (%)	*n* = 401 (%)	<0.001
No	166 (60.4)	115 (91.3)	281 (70.1)	
Patient no longer hospitalised	89 (32.4)	7 (5.6)	96 (23.9)	
Yes	20 (7.3)	4 (3.2)	24 (6.0)	

AFP—antifungal prophylaxis, PPI—proton pump inhibitor.

**Table 6:** Follow-up: patient status 15 days after AFP, neutropenia periods and deaths.

Characteristics	Switch to a Non-Prophylactic Treatment			
	No (S1)	Yes (S2)	Total	*p*-Value
Signs/symptoms of infection at day 15:	*n* = 268 (%)	*n* = 119 (%)	*n* = 387 (%)	<0.001
Yes	2 (0.7)	20 (16.8)	22 (5.7)	
Change of hospitalisation conditions	*n* = 268 (%)	*n* = 119 (%)	*n* = 387 (%)	<0.001
No	76 (28.4)	95 (79.8)	171 (44.2)	
Yes, patient no longer hospitalised	167 (62.3)	20 (16.8)	187 (48.3)	
Yes	25 (9.3)	4 (3.4)	29 (7.5)	
Profound neutropenia periods during the entire follow up period				
Neutropenia periods, *n* = number of patients	*n* = 249	*n* = 121	*n* = 370	0.021
Mean (SD)	1.0 (0.3)	1.2 (0.8)	1.1 (0.5)	
Median	1.0	1.0	1.0	
Range	1.0–5.0	1.0–6.0	1.0–6.0	
Duration (days), *n* = number of neutropenia periods	*n* = 254	*n* = 139	*n* = 393	0.388
Mean (SD)	22.7 (16.7)	24.2 (16.6)	23.3 (16.6)	
Median	21.0	22.0	22.0	
Range	2.0–172	1.0–162	1.0–172	
Deaths				
Time between inclusion and death (days)	*n* = 11	*n* = 9	*n* = 20	0.634
Mean (SD)	62.6 (56.1)	51.4 (44.8)	57.6 (50.3)	
Median	30.0	19.0	28.5	
Range	15.0–184	11.0–122	11.0–184	
Signs/symptoms of infection:	*n* = 11 (%)	*n* = 9 (%)	*n* = 20 (%)	0.160
Yes	2 (18.2)	5 (55.6)	7 (35.0)	
Death linked to IFD	*n* = 11 (%)	*n* = 9 (%)	*n* = 20 (%)	0.074
Yes	-	3 (33.3)	3 (15.0)	

AFP: antifungal prophylaxis; IFD: invasive fungal disease.

**Table 7:** Multivariate logistic regression of significantly different parameters at baseline, AFP initiation, end of AFP treatment and 15-day follow-up.

Baseline and AFP initiation			
**Factor**	**Modalities**	**Odds ratio**	**95% CI**
Cytogenetics/molecular biology of prognosis	Favourable *versus* Unknown	1.411	0.426–4.676
	Intermediate *versus* Unknown	2.669	0.844–8.444
	Unfavourable *versus*. Unknown	3.639	1.129–11.723
Previous chemotherapy	No *versus* Yes	2.033	1.151–3.589
Duration between start of 1st chemotherapy and inclusion	Increasing duration between ongoing chemotherapy and inclusion (in days)	1.064	1.007–1.124
Loading dose	No *versus* Yes	1.811	1.116–2.938
End of AFP treatment and 15-day follow-up			
**Factor**	**Modalities**	**Odds ratio**	**95% CI**
Patient alimentation at the end of AFP	Patient cannot eat, and oral medication intake is impossible *versus* Normal food	5.031	0.907–27.905
	Patient cannot eat, but oral medication intake is possible *versus* Normal food	4.983	1.155–21.504
	Protected food *versus* Normal food	4.741	1.392–16.147
	Sterile food *versus* Normal food	2.131	0.596–7.613
Change of hospitalisation conditions at the end of AFP	No *versus* Yes, the patient is no longer hospitalized	5.452	1.851–16.060
	Yes *versus* Yes, the patient is no longer hospitalized	1.515	0.166–13.828
Signs and symptoms of infection at day 15	Yes *versus* No	7.286	1.275–41.648
Change of hospitalisation conditions at 15 days	No *versus* Yes, the patient is no longer hospitalized	7.370	3.776–14.383
	Yes *versus* Yes, the patient is no longer hospitalized	1.083	0.303–3.870
Number of neutropenia periods	Increasing number of neutropenia periods	9.939	3.420–28.881

AFP: antifungal prophylaxis.
